# Visceral Leishmaniasis: Integrated In Silico Screening of Djiboutian Medicinal Plant Phytoconstituents Targeting *Leishmania donovani* and *Leishmania infantum*

**DOI:** 10.3390/ph19050730

**Published:** 2026-05-06

**Authors:** Fatouma Mohamed Abdoul-Latif, Amal Bouribab, Houda Mohamed, Lamiae El Bouamri, Bouchra Rossafi, Fatimazahra Guerguer, Imane Yamari, Yahya Ali Ismael, Pannaga Pavan Jutur, Samir Chtita

**Affiliations:** 1 Medicinal Research Institute, Center for Research and Study of Djibouti, Djibouti City BP 486, Djibouti; abdoulhouda@yahoo.fr (H.M.); soumade2015@gmail.com (Y.A.I.); 2 Laboratory of Analytical and Molecular Chemistry, Faculty of Sciences Ben M’Sick, Hassan II University of Casablanca, Casablanca 20670, Morocco; bouribabamal@gmail.com (A.B.); elbouamrilamiae14@gmail.com (L.E.B.); bouchrarossafi7@gmail.com (B.R.); guerguerfatimazahra@gmail.com (F.G.); yamariimane86@gmail.com (I.Y.); 3 CHU Peltier Hospital of Djibouti, Djibouti City P.O. Box 2123, Djibouti; 4 International Centre for Genetic Engineering and Bio-Technology, Aruna Asaf Ali Marg, New Delhi 110067, India; pavan.jutur@icgeb.org

**Keywords:** visceral leishmaniasis, *Leishmania donovani*, *Leishmania infantum*, medicinal plants, phytoconstituents, molecular docking, molecular dynamics simulation, ADMET prediction, drug discovery, in silico screening

## Abstract

**Objectives**: Visceral leishmaniasis (VL), caused by *Leishmania donovani* and *Leishmania infantum*, is a life-threatening neglected tropical disease, particularly in endemic regions such as Djibouti. Current therapies are constrained by toxicity, high cost, and limited availability, highlighting the urgent need for safe, effective, and affordable alternatives. This study aimed to identify novel antileishmanial candidates from Djiboutian medicinal plants using an integrated in silico approach. **Methods**: A total of 136 phytoconstituents isolated from local medicinal plants were screened via molecular docking against validated protein targets (6UAK and 2JK6). Promising candidates were further analyzed for interaction patterns, drug-likeness according to Lipinski’s Rule of Five, and ADMET properties. Molecular dynamics (MD) simulations over 100 ns were performed to assess the structural stability of selected protein–ligand complexes. **Results**: Compound C41 emerged as a leading candidate, showing binding affinities of −8.3 kcal/mol and −7.5 kcal/mol toward 6UAK and 2JK6, respectively, comparable to reference drugs. Interaction analysis revealed stable hydrogen bonds and hydrophobic contacts within the catalytic sites. Drug-likeness assessment confirmed compliance with Lipinski’s Rule, while ADMET predictions indicated high intestinal absorption and favorable safety profiles for several candidates. MD simulations corroborated the structural stability of the 2JK6-C41 complex throughout the 100 ns trajectory. **Conclusions**: These findings underscore Djiboutian medicinal plants as a valuable source of potential antileishmanial leads. Among them, Compound C41 represents a promising candidate for future experimental validation, supporting the development of innovative, safe, and cost-effective therapies against visceral leishmaniasis.

## 1. Introduction

Visceral leishmaniasis (VL), also known as kala-azar, is the most severe form of leishmaniasis and remains one of the most significant neglected tropical diseases worldwide [1]. Caused by protozoan parasites from the genus *Leishmania*, mostly *Leishmania donovani* and *Leishmania infantum*, VL my cause fatality if left untreated and continues to affect low- and middle-income countries unproportionally where access to timely diagnosis and effective treatment is often limited [2]. Transmission occurs through the bite of infected female phlebotomine sand flies, which allow the disease to spread in endemic areas [3].

Visceral leishmaniasis (VL), also known as kala-azar, is the most severe form of leishmaniasis and remains one of the most significant neglected tropical diseases worldwide [1,4]. Caused by protozoan parasites of the genus Leishmania, mainly *Leishmania donovani* and *Leishmania infantum*, VL can be fatal if left untreated and disproportionately affects populations in low- and middle-income countries with limited access to diagnosis and treatment [5,6]. Transmission occurs through the bite of infected female phlebotomine sand flies, enabling the persistence of the disease in endemic regions [3].

Serological diagnostic tools such as the direct agglutination test (DAT) and the rK39 rapid diagnostic test are widely used due to their high sensitivity and ease of use [4]. However, these methods present limitations, including reduced accuracy in immunocompromised individuals, decreased specificity in endemic settings, and the inability to distinguish between active and past infections [7].

According to the Institut National de Santé Publique de Djibouti, the first reported cases of VL emerged in 2015 in the Obock region, particularly in Assassan, indicating active transmission in this area [5]. These findings highlight the continued circulation of VL in the country and emphasize the urgent need to strengthen epidemiological surveillance, improve early diagnosis, and implement targeted public health interventions.

Treatment of VL is lifesaving and must be initiated promptly to prevent fatal outcomes [8]. Current therapeutic options include liposomal amphotericin B, amphotericin B deoxycholate, pentavalent antimonials, miltefosine, and paromomycin [9]. Liposomal amphotericin B is considered the treatment of choice due to its high efficacy and improved safety profile; however, its high cost, requirement for cold-chain storage, and limited accessibility restrict its use in endemic regions [9]. Additionally, issues such as drug toxicity, prolonged treatment regimens, emerging resistance, and poor patient adherence continue to complicate disease management.

Despite significant advances in understanding Leishmania biology, host–parasite interactions, and immunopathogenesis, VL remains underfunded and insufficiently addressed in terms of innovation and therapeutic development [4]. Although progress in molecular biology and genomics has enhanced our knowledge of parasite diversity and pathogenic mechanisms, these advances have not yet fully translated into accessible diagnostics, safer treatments, or effective vaccines [10,11]. Strengthening surveillance systems, expanding access to healthcare, and adopting integrated approaches are essential to reducing disease burden.

Post-kala-azar dermal leishmaniasis (PKDL) is a recognized complication following VL treatment and plays a role in maintaining parasite reservoirs and sustaining transmission cycles [8]. These challenges underscore the urgent need to identify alternative therapeutic strategies that are safer, more accessible, and adapted to endemic settings such as Djibouti [12,13].

In this context, computational approaches have emerged as powerful tools in modern drug discovery, particularly for neglected diseases such as VL [14]. In silico methods, including molecular docking, virtual screening, pharmacokinetic (ADMET) prediction, and molecular dynamics simulations, enable rapid identification and optimization of bioactive compounds while reducing experimental cost and time [15]. Molecular docking provides insights into ligand–target interactions and binding affinities within active sites, whereas ADMET analysis evaluates pharmacokinetic and toxicity profiles at early stages of drug development. Furthermore, molecular dynamics simulations offer a dynamic perspective on protein–ligand stability under physiological conditions, improving the reliability of docking predictions [15]. Together, these computational techniques represent a cost-effective and efficient strategy for accelerating the discovery of novel antileishmanial agents [14].

The selected targets, 6UAK and 2JK6, were chosen based on their essential roles in parasite survival. Trypanothione reductase (2JK6) is crucial for maintaining redox homeostasis and protecting the parasite against oxidative stress, while enzymes involved in key metabolic pathways represent attractive therapeutic targets [10,11]. Targeting such proteins may disrupt critical biological processes and enhance therapeutic efficacy.

Medicinal plants constitute a valuable source of bioactive compounds with potential antileishmanial properties [15,16]. Traditionally used in various regions for their antimicrobial, anti-inflammatory, and antiparasitic effects, these natural resources offer promising alternatives for drug discovery. However, many geographical areas, including Djibouti, remain underexplored in this context. Therefore, the present study aims to investigate phytocompounds derived from medicinal plants using an integrated in silico approach combining molecular docking, ADMET prediction, and molecular dynamics simulations to identify potential candidates for VL treatment.

## 2. Results

### 2.1. Docking Validation

**Figure 1:**
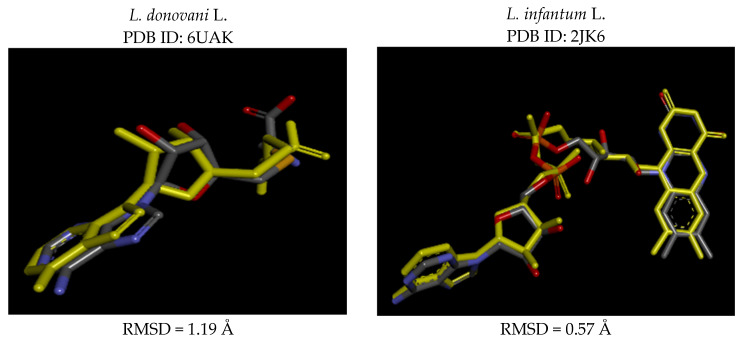
Three-dimensional representation of co-ligand superposition before (yellow) and after docking.

Validation of the reliability of the method was achieved through co-ligand anchoring tests on the 6UAK and 2JK6 receptor proteins. The process allowed us to track co-ligand positioning which we used to determine RMSD values. The method requires RMSD values to remain below 2 Å for its validation process [17,18]. Our results showed an RMSD measurement of 1.19 Å for 6UAK and 0.57 Å for 2JK6, which demonstrated effective structural alignment based on the results shown in Figure 1. The findings demonstrate that our docking method produces reliable results.

By selecting both 6UAK and 2JK6, we aimed to investigate compounds that could interfere with distinct yet essential biological pathways, thereby increasing the likelihood of discovering effective antileishmanial agents.

### 2.2. Docking Analyses

After validation of the docking protocol, virtual screening was performed to identify potential candidate molecules. The results presented in Table 1 indicate that compound C41 exhibited a favorable binding score toward the 2JK6 target, with a docking energy of −7.5 kcal/mol. Two reference drugs, liposomal amphotericin B and miltefosine, were included for comparison, showing binding energies of −8.6 kcal/mol and −7.3 kcal/mol, respectively.

Regarding *Leishmania donovani*, a total of 34 compounds demonstrated potential binding to the 6UAK target (Table S1), with docking scores comparable to or better than that of liposomal amphotericin B (−7.3 kcal/mol). Among these, compound C41 showed the most favorable binding affinity (−8.3 kcal/mol), suggesting a strong potential interaction with the target protein.

**Figure 2:**
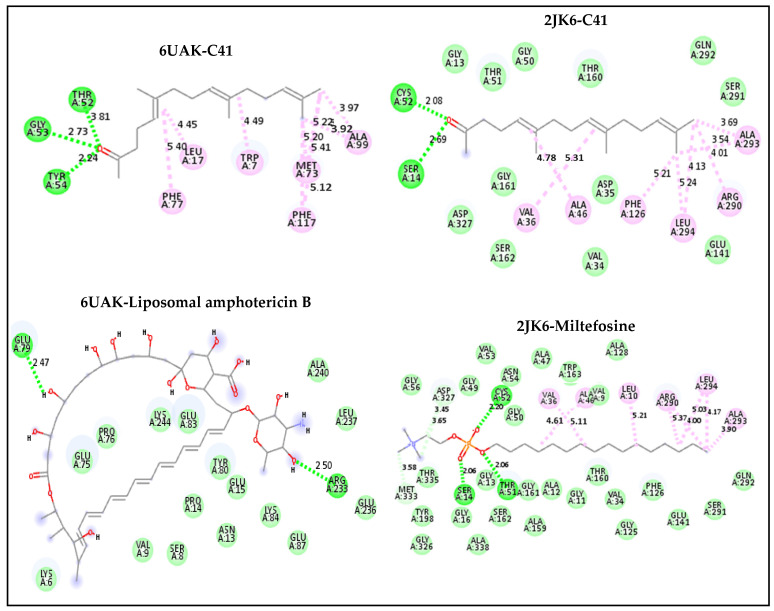
2D interaction maps of C41 and drug reference with 6UAK and 2JK6.

The favorable binding affinity predicted for compound C41 may be attributed to the presence of hydrophobic interactions and hydrogen bonds contributing to the stabilization of the ligand–protein complex (Figure 2). Within the complex formed with the 6UAK protein, compound C41 was predicted to form hydrogen bonds with residues THR52, GLY53, and TYR54, which may contribute to ligand stabilization within the active site. Additionally, hydrophobic interactions with residues LEU17, TRP7, MET73, ALA99, PHE77, and PHE117 were identified, potentially supporting the anchoring of the ligand within the binding cavity.

In comparison, the 6UAK–liposomal amphotericin B complex exhibited a different interaction pattern, involving hydrogen bonds with GLU79 and ARG233. For the 2JK6-C41 complex, the ligand was predicted to be stabilized by multiple hydrophobic interactions involving residues VAL36, ALA46, PHE126, LEU294, ARG290, and ALA293, similar to those observed with the reference drug miltefosine, with slight variations (e.g., PHE126 replaced by LEU10). Furthermore, hydrogen bonds with residues CYS52 and SER14 were identified, which may play a role in ligand orientation and stability within the binding site.

Overall, these interactions suggest that compound C41 may exhibit favorable binding properties toward the active sites of both protein targets and could represent a promising candidate for further investigation.

### 2.3. ADMET Prediction

The 34 selected ligands were evaluated for their compliance with Lipinski’s rule of five, an important criterion for predicting oral bioavailability [19]. As shown in Table 2, all studied compounds complied with these rules, suggesting favorable drug-likeness and potential for oral administration.

Regarding the predicted toxicity assessment, the results indicated that three compounds (C22, C44, and C90) did not show any predicted toxic effects, highlighting their potential as candidates for further pharmacological investigation. In contrast, several other compounds were predicted to present a risk of skin toxicity. However, two exceptions (C94 and C136) did not show predicted skin toxicity but were associated with potential mutagenicity according to the Ames test, indicating a possible genotoxic risk. Furthermore, compound C72 was predicted to exhibit hepatotoxicity, which may limit its therapeutic potential and warrants further investigation.

The predicted absorption profiles suggested high intestinal absorption for all studied compounds, with estimated absorption rates exceeding 90%, indicating potential suitability for oral delivery (Table 3). Most compounds showed low predicted skin permeability, except for compound C79, which exhibited relatively higher permeability (log Kp = −4.01).

In terms of distribution, the predicted values ranged from 0 to 1.11, suggesting moderate to favorable distribution profiles among the studied compounds. Notably, compound C133 showed the highest predicted distribution capacity. Furthermore, the majority of studied compounds possess the ability to penetrate the blood–brain barrier (BBB), suggesting that these substances could potentially affect central nervous system functions. The molecule C79 showed a moderate ability to cross this barrier. However, in the context of visceral leishmaniasis, which primarily affects peripheral organs such as the liver and spleen, BBB penetration is not necessarily required and may even be undesirable due to the potential risk of central nervous system side effects. Therefore, compounds with limited BBB permeability may present a more favorable safety profile. Concerning metabolism, the study found that none of the tested molecules act as substrates of the CYP2D6 metabolic pathway, which may reduce variability in drug metabolism associated with this enzyme. However, several compounds (C41, C22, C94, C136, C70, C63, C83, C78, C90, C26, C31, C38, and C133) showed interactions with the CYP1A2 metabolic pathway. In addition, the majority of molecules exhibited inhibitory activity against CYP1A2, particularly C41, C22, C40, C72, C94, C136, C70, C90, C31, and C133. From a pharmacological perspective, inhibition of cytochrome P450 enzymes, especially CYP1A2, may lead to potential drug–drug interactions and metabolic disturbances, which could limit the clinical applicability of these compounds. Similarly, the inhibitory activity observed for CYP2C19 (C70, C98, and C49) and CYP2C9 (C98) suggests possible metabolic liabilities that should be carefully considered. Therefore, compounds with lower CYP inhibition profiles may be more suitable candidates for further drug development [19]. Finally, the total clearance values for all studied molecules showed prediction results that ranged from 0.01 log mL/min/kg for molecule C72 to 1.81 log mL/min/kg for molecule C81, which demonstrated that these compounds exhibit highly different elimination rates in human body systems.

Among the studied compounds, C41 was identified as the most promising candidate due to its strong binding affinity against both target proteins. In terms of ADMET properties, C41 exhibited several favorable characteristics, including an appropriate molecular weight, absence of predicted mutagenicity (AMES test), no hepatotoxicity, and no significant risk of cardiotoxicity (hERG inhibition). However, some potential liabilities were also identified. The relatively high lipophilicity (LogP = 5.77) may affect solubility and bioavailability. In addition, the predicted ability to cross the blood–brain barrier and the positive skin sensitization may raise safety concerns, particularly in the context of visceral leishmaniasis where central nervous system exposure is not required. Furthermore, the predicted inhibition of cytochrome P450 enzymes suggests a possible risk of drug–drug interactions. Although C41 demonstrated promising pharmacokinetic and safety profiles, some liabilities were identified, including predicted blood–brain barrier (BBB) penetration and skin sensitization. For the treatment of visceral leishmaniasis, BBB permeability is not required and may lead to undesired central nervous system exposure. This property is likely related to the relatively high lipophilicity of Compound C41 (LogP = 5.77). In future lead optimization, this limitation could be addressed by reducing lipophilicity and increasing molecular polarity through the introduction of polar functional groups or hydrogen bond donors/acceptors. In addition, the predicted skin sensitization potential may be associated with the presence of reactive functional groups. This issue could be mitigated by structural modifications such as bioisosteric replacement or reduction in electrophilic reactivity. Overall, these findings suggest that while C41 presents certain ADMET limitations, these can be rationally optimized without compromising its antileishmanial potential.

### 2.4. MD Simulations Analysis

**Figure 3:**
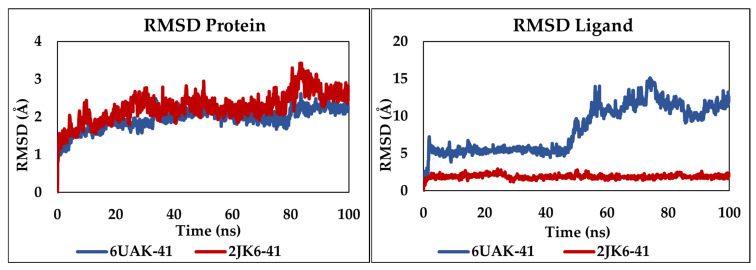
RMSD protein and ligand plots for the simulated complexes 6UAK-C41 and 2JK6-C41.

Molecular dynamics simulations were carried out over 100 ns to evaluate the stability of the complexes formed between the selected molecule C41 and the two proteins 6UAK and 2JK6. The results are illustrated in Figure 3.

For the protein RMSD, both complexes 6UAK-C41 and 2JK6-C41 exhibited an initial decrease in RMSD values during the first few nanoseconds, corresponding to the equilibration phase of the system. After approximately 20 ns, the RMSD values fluctuating around ~1.5–3.0 Å, suggesting minimal conformational deviations throughout the simulation. A minor increase observed between 80 and 84 ns for 2JK6-C41 complex up to 3.5 Å. However, these deviations remained within an acceptable range, indicating that ligand binding does not significantly disrupt the overall structural integrity of the target enzyme.

For the ligand RMSD, 2JK6-C41 complex exhibited remarkably low RMSD fluctuations, approximately ~1.5–2.0 Å throughout the simulation, indicating a great stability and that the ligand C41 remained strongly anchored within the active site of the protein without undergoing significant conformational displacement. In contrast, the 6UAK-C41 complex exhibited only moderate stability during the initial phase of the simulation. After approximately 50 ns, a significant increase in the ligand RMSD was observed, reaching values between ~10 and 14 Å. Such high fluctuations indicate pronounced ligand mobility and suggest a loss of positional stability within the binding pocket. This behavior may reflect partial unbinding events, an unstable binding pose, or insufficient complementarity between the ligand and the 6UAK active site.

**Figure 4:**
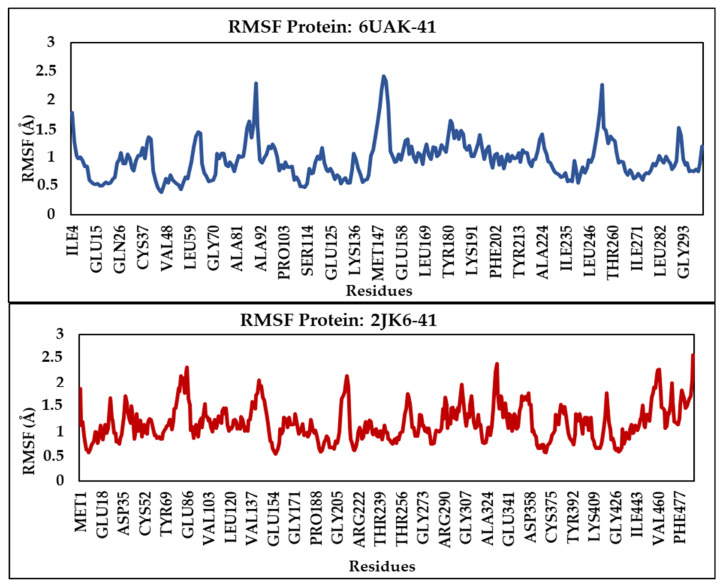
RMSF protein plots for the simulated complexes 6UAK-C41 and 2JK6-C41.

The protein RMSF analysis provides insights into the flexibility and dynamic behavior of individual residues during the simulation [20]. Low RMSF values indicate that the residues remain stable and exhibit minimal fluctuations, suggesting structural stability in these regions. The results of this analysis are presented in Figure 4. Most residues in the 6UAK-C41 complex exhibit low RMSF values, indicating that they remain relatively stable during the simulation. Although Leu90, Pro151, and Leu252 show the higher fluctuations, these remain moderate and do not compromise the overall stability of the protein–ligand complex. Similarly, in the 2JK6-C41 complex, all residues display low fluctuations, with RMSF values not exceeding 2.5 Å, demonstrating that the protein maintains high structural stability throughout the simulation.

Notably, molecular dynamics simulations revealed distinct behaviors between the two targets, with the 2JK6-C41 complex showing a relatively stable profile, while the 6UAK-C41 system exhibited increased ligand mobility and significant displacement, as reflected by the higher ligand RMSD values observed during the simulation.

**Figure 5:**
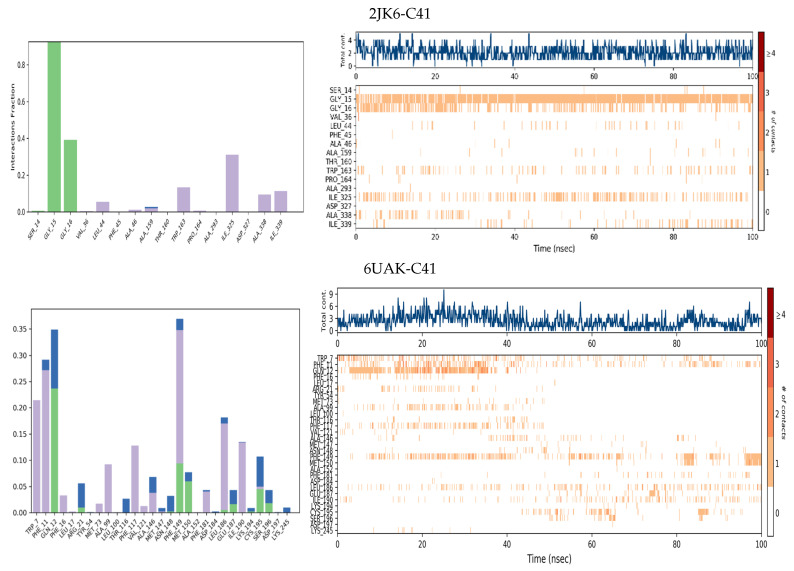
Protein–ligand contact histogram/timeline for the simulated complexes. Green bars represent hydrogen bonds, purple bars indicate hydrophobic interactions, and blue bars show water bridges.

Figure 5 illustrates the interactions established between the ligand 41 and proteins residues throughout the simulation. For 2JK6-C41 complex, residues GLY15 and GLY16 exhibit the highest interaction fractions close to 0.9 and 0.4, respectively, indicating that these residues are the most frequently involved in hydrogen bonds with the ligand. Other residues, such as ILE325, show hydrophobic interactions with moderate fraction (~0.3), while several residues exhibit only minor fractions. The timeline showed that GLY15 and GLY16 maintain persistent interactions throughout most of the simulation, reflecting strong and stable contacts, whereas residues like LEU44, TRP163, and ASP327 display intermittent interactions. This pattern confirms that molecule C41 forms stable binding with key residues while allowing some flexibility in less critical regions of the binding site.

For the 6UAK-C41 complex, several residues, such as TRP7, PHE11, GLN12, and PHE149, exhibited both hydrogen bonding and hydrophobic interactions, accompanied by water bridges. However, the interaction fractions were generally moderate. The protein–ligand timeline indicates that these interactions were intermittent and not maintained throughout the simulation, which explains the observed increase in the ligand RMSD for this complex.

The radius of gyration (Rg) is a key parameter in molecular dynamics simulations, defined as the mass-weighted root mean square distance of a set of atoms from their common center of mass. It provides essential insights into the overall compactness and structural stability of a protein, as well as global conformational changes that may occur during the simulation. Variations in Rg values can therefore reflect folding–unfolding events or structural rearrangements within protein–ligand complexes.

**Figure 6:**
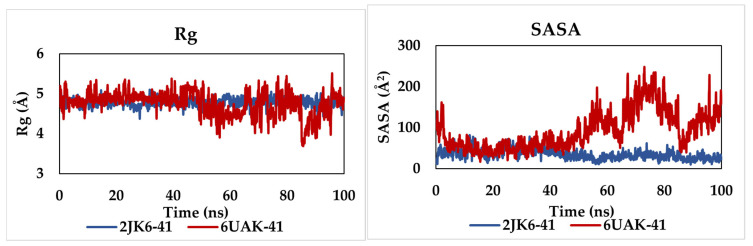
Rg and SASA plots for the simulated complexes.

As illustrated in Figure 6, the Rg values remained relatively stable throughout the simulation for both systems, with average values of 4.79 ± 0.08 Å and 4.76 ± 0.23 Å for the 2JK6-C41 and 6UAK-C41 complexes, respectively. The low fluctuations observed suggest that both complexes maintain a compact structure and exhibit a high degree of conformational stability over the simulation time.

To further investigate the conformational behavior and solvent exposure of the complexes, solvent-accessible surface area (SASA) analysis was performed (Figure 6). SASA is a useful descriptor for evaluating the extent to which the protein surface is accessible to solvent molecules, and thus provides information about folding, structural flexibility, and ligand-induced conformational changes. The average SASA values for the 2JK6-C41 and 6UAK-C41 systems were 35.44 ± 9.89 Å^2^ and 93.09 ± 39.91 Å^2^, respectively. The higher SASA value observed for the 6UAK-C41 complex indicates a greater exposure to the solvent, which may reflect increased flexibility or a more open conformation compared to the 2JK6-C41 system. Conversely, the lower SASA values for 2JK6-C41 suggest a more compact and less solvent-exposed structure, consistent with its stable Rg profile.

### 2.5. MM/GBSA Analysis

The binding free energy values provide important insights into the stability of the ligand–protein complexes [21]. As a general rule, more negative binding free energy (ΔG) values indicate stronger interactions [22]. The calculated ΔG values for the studied systems indicate that compound 41 exhibits strong and favorable interactions with 2JK6 (Table 4). The 2JK6-C41 complex shows a ΔG of −65.33 kcal/mol, while the 6UAK-C41 complex presents a value of −55.00 kcal/mol. Notably, the more negative value obtained for the 2JK6-C41 system suggests a higher binding affinity and greater stability compared to 6UAK-C41. Importantly, these findings are in good agreement with the MD simulation results, which also demonstrated enhanced stability for the 2JK6-C41 complex over the simulation time.

To assess the robustness and reproducibility of the observed dynamic behavior, additional independent MD simulations were performed for the 6UAK-C41 and 2JK6-C41 complex under identical simulation conditions. The replicate runs consistently reproduced the previously observed instability of 6UAK-C41, as reflected by comparable RMSD profiles, residue fluctuation patterns (RMSF), and protein–ligand interaction. The detailed results of the replicate simulations are provided in the Supplementary Material (Figures S1 and S2).

## 3. Discussion

Among the compound library derived from medicinal plants collected in Djibouti, which remain largely unexplored for antileishmanial applications, Farnesyl acetone, identified from *Lavandula coronopifolia* L., exhibited a promising predicted antileishmanial potential, particularly against the 2JK6 target. This compound showed favorable docking scores, stable behavior during molecular dynamics simulations, and acceptable predicted pharmacokinetic properties.

Farnesyl acetone and related phytochemicals derived from Djiboutian medicinal plants have been reported to exhibit significant antioxidant and cytotoxic activities against various cancer cell lines, including HCT-116, MCF-7, and HepG2 [23]. In addition, terpenoid compounds structurally related to Farnesyl acetone have demonstrated diverse biological activities, including insecticidal effects against *Plutella xylostella* [24]. These findings support the potential of such compounds as bioactive scaffolds and justify further investigation of their predicted antileishmanial activity.

These results highlight the importance of exploring under-investigated natural resources and suggest that medicinal plants from Djibouti may represent a promising reservoir of bioactive compounds for the development of new therapeutic agents against visceral leishmaniasis.

Despite these promising findings, several limitations should be acknowledged. Molecular docking provides only a qualitative estimation of binding affinity, and small differences in docking scores should be interpreted with caution. Molecular dynamics simulations supported the stability of ligand–protein complexes; however, the limited simulation time and lack of replicate runs may affect the robustness of the results. In addition, ADMET predictions are based on computational models and may not fully reflect in vivo pharmacokinetic behavior or toxicity. Furthermore, potential synergistic effects between phytoconstituents were not evaluated, although they may significantly contribute to biological activity in natural extracts. Therefore, experimental validation remains essential to confirm the predicted antileishmanial activity and pharmacological properties of the identified compounds.

## 4. Material and Methods

### 4.1. Data Sources

The studied database was compiled from recent literature, comprising 136 phytoconstituents isolated from seven medicinal plants collected from different regions of Djibouti [25,26,27,28]. The 2D and 3D structures were generated using ChemOffice software (version 2020). These structures were then optimized using the MMFF94 force field and the steepest descent algorithm via the Avogadro molecular software (version 2022), before being saved in PDB files. Table S1 (Supporting Information) provides the structures of the studied compounds along with information such as the plant name, compounds name and SMILES and region of origin.

### 4.2. Molecular Docking

In silico studies provide researchers with a useful method which allows them to study molecular complex interaction mechanisms [29]. In the present study, a molecular docking analyses was used to examine a collection of 136 compounds which they tested for their ability to treat VL. To better understand ligand-target interactions and find potential candidates with high binding affinities toward the chosen biological target, the docking simulations were carried out using AutoDock Vina software (version 1.5.7) [30].

We particularly focused on the inhibition of the *Leishmania donovani* and *Leishmania infantum*. To achieve this, we downloaded the 3D structures of the proteins responsible for these two activities from the RCSB PDB server (https://www.rcsb.org/) (accessed on 2 February 2026), using PDB ID: 6UAK for *L. donovani* L. and PDB ID: 2JK6 for *L. infantum* L., with a resolution of 2.1 and 2.95 Å respectively [31,32]. Subsequently, the receptors were prepared for docking studies by removing all co-crystallized ligands, solvent molecules, and non-essential metal ions. We also introduced polar hydrogen atoms and Gasteiger charges and addressed any missing atoms and residues. The ligands were subsequently prepared and converted into the AutoDock ligand format (PDBQT) to enable docking simulations [33]. The binding site for docking was defined based on the position of the co-crystallized ligand in the original PDB structures. For 6UAK, the docking grid was centered on the cofactor-binding cavity defined by the co-crystallized S-adenosyl-L-homocysteine. This region includes key residues such as Thr52, His115, and Phe117, which are involved in ligand binding and catalytic activity (Table 5). For 2JK6, the grid was positioned on the FAD-binding pocket, encompassing important residues including Ser14, Gly15, Tyr198, Gly127, and Asp327 that contribute to the formation of the active redox site essential for the enzyme function in *Leishmania infantum*. The grid box parameters, including center coordinates and size, are summarized in Table S2 (Supporting Information). The docking protocol was validated by redocking the co-crystallized ligand, confirming the reliability of the method.

### 4.3. Drug Likeness and ADMET Prediction

Predict of pharmacokinetic properties plays a crucial role in the therapeutic validation of the compounds we have selected. These encompass essential parameters such as absorption, distribution, metabolism, excretion, and toxicity (ADMET) [34]. Using canonical SMILES, we employed the pkCSM web server (https://biosig.lab.uq.edu.au/pkcsm/) (accessed on 6 February 2026) to evaluate the compliance of the selected compounds with Lipinski’s Rule of Five (Ro5) in order to predict their oral bioavailability [35]. Additionally, a comprehensive ADMET analysis was performed to provide essential insights into the predicted in vivo pharmacological behavior of the investigated compounds. This integrated computational strategy enables a rigorous screening process, facilitating the identification of compounds that meet both therapeutic potential and bioavailability requirements, while strictly adhering to common pre-clinical safety standards.

### 4.4. Molecular Dynamics Simulation

Molecular dynamics simulations represent a powerful approach in biochemistry, pharmacology, and materials science, as they provide detailed insights into molecular interactions and dynamic processes that are often difficult to investigate experimentally [36]. In the present study, molecular dynamics simulations were performed to evaluate the structural stability of compound C41, which demonstrated strong activity toward both receptors 6UAK and 2JK6. The simulations were conducted using Desmond, a cornerstone of the Schrödinger Release 2020-3: Maestro software suite, for a simulation time of 100 ns [37]. Prior to the simulation, the receptor structures were prepared by removing overlapping water molecules, followed by solvation of the system using crystallographic water molecules modeled with the TIP3P water model under cubic periodic boundary conditions with a buffer distance of 10 Å [38]. System neutralization was achieved by adding appropriate Na^+^ or Cl^−^ counterions to maintain a physiological salt concentration of 0.15 M. The OPLS3e force field was then employed to assign parameters for the protein. Temperature and pressure conditions were stabilized using an isothermal-isobaric ensemble with the Nose–Hoover thermostat and Martyna-Tobias-Klein barostat, which are the default protocols [39]. Subsequently, the systems underwent NVT equilibration for 1 ns, followed by NPT equilibration at 300 K and 1.01 bar for a simulation duration of 100 ns. Throughout the simulation, trajectories were recorded at intervals of 100 ps, generating approximately 1000 frames.

### 4.5. MM-GBSA Calculation

The MM/GBSA (Molecular Mechanics/Generalized Born Surface Area) method, as implemented in the Schrodinger software (version 2020-3), was employed to calculate the binding free energy (ΔG) of the protein–ligand complexes. In this study, ΔG was estimated based on the final structure of the complex extracted from the MD simulation [40]. This method provides insights into the thermodynamics of ligand binding and offers an efficient approach to analyze the energetic interactions between proteins and ligands. The following equation is used to determine the binding free energy (ΔG) of a protein–ligand system:

∆G=Gcomplex−(Gprotein+Gligand)

## 5. Conclusions

This study highlights the potential of Djiboutian medicinal plants as a source of novel antileishmanial compounds. Using an integrated computational approach, farnesyl acetone (compound C41), isolated from *Lavandula coronopifolia* L., was identified as a promising candidate with potential activity against *Leishmania donovani* and *Leishmania infantum*, exhibiting favorable binding affinity and predicted pharmacokinetic properties. However, molecular dynamics simulations revealed differential stability profiles, with a more stable behavior observed for the 2JK6 complex compared to the 6UAK system. These findings underscore the usefulness of in silico screening approaches in the early-stage identification of potential bioactive compounds. However, it is important to note that these results are based on computational predictions and require further validation through in vitro and in vivo studies. Such investigations are necessary to confirm the biological activity and therapeutic potential of the identified compounds and to support their possible development as alternative treatments for visceral leishmaniasis.

## Figures and Tables

**Table 1:** Docking scores of the top 10 ligands against 6UAK and 2JK6.

*L. donovani* L.		*L. infantum* L.	
Compound	Score (kcal/mol)	Compound	Score (kcal/mol)
C41	−8.3	C41	−7.5
C22	−8.2	C49	−7.2
C27	−8.1	C75	−7.2
C40	−8.1	C27	−7.1
C33	−8	C72	−7.1
C72	−8	C34	−7.1
C94	−8	C70	−7
C136	−7.9	C47	−7
C110	−7.8	C61	−6.9
C24	−7.7	C30	−6.9
Liposomal amphotericin B	−7.3	Liposomal amphotericin B	−8.6
Miltefosine	−6.1	Miltefosine	−7.3

**Table 2:** Prediction of oral bioavailability and toxicology of selected compounds.

	MW	LOGP	Hb a	Hb d	AMES Toxicity	hERG I Inhibitor	hERG II Inhibitor	Hepatotoxicity	Skin Sensitization
C41	262.44	5.77	1.00	0.00	No	No	No	No	Yes
C22	204.36	4.27	0.00	0.00	No	No	No	No	No
C27	204.36	4.89	0.00	0.00	No	No	No	No	Yes
C40	220.36	3.84	1.00	1.00	No	No	No	No	Yes
C33	204.36	5.18	0.00	0.00	No	No	No	No	Yes
C72	190.20	2.02	3.00	0.00	No	No	No	Yes	Yes
C94	204.36	4.27	0.00	0.00	Yes	No	No	No	No
C136	204.36	4.27	0.00	0.00	Yes	No	No	No	No
C110	222.37	4.06	1.00	1.00	No	No	No	No	Yes
C24	204.36	4.89	0.00	0.00	No	No	No	No	Yes
C44	206.37	4.49	0.00	0.00	No	No	No	No	No
C51	222.37	4.40	1.00	1.00	No	No	No	No	Yes
C68	222.37	4.40	1.00	1.00	No	No	No	No	Yes
C70	232.32	3.52	2.00	0.00	No	No	No	No	Yes
C98	222.37	4.06	1.00	1.00	No	No	No	No	Yes
C34	204.36	4.58	0.00	0.00	No	No	No	No	Yes
C49	222.37	4.23	1.00	1.00	No	No	No	No	Yes
C63	204.36	4.58	0.00	0.00	No	No	No	No	Yes
C65	204.36	4.89	0.00	0.00	No	No	No	No	Yes
C79	220.36	4.41	1.00	0.00	No	No	No	No	Yes
C81	204.36	5.20	0.00	0.00	No	No	No	No	Yes
C83	220.36	4.44	1.00	0.00	No	No	No	No	Yes
C61	166.26	2.79	1.00	0.00	No	No	No	No	Yes
C78	204.36	4.73	0.00	0.00	No	No	No	No	Yes
C90	204.36	4.42	0.00	0.00	No	No	No	No	No
C99	220.36	3.84	1.00	1.00	No	No	No	No	Yes
C104	196.29	3.24	2.00	0.00	No	No	No	No	Yes
C25	204.36	4.73	0.00	0.00	No	No	No	No	Yes
C26	204.36	4.73	0.00	0.00	No	No	No	No	Yes
C31	204.36	4.73	0.00	0.00	Yes	No	No	No	Yes
C38	222.37	3.94	1.00	1.00	No	No	No	No	Yes
C133	202.34	4.63	0.00	0.00	Yes	No	No	No	Yes
C28	204.36	5.04	0.00	0.00	No	No	No	No	Yes
C35	204.36	4.73	0.00	0.00	No	No	No	No	Yes

MW: Molecular weight; Hb a: Num. H-bond acceptors; Hb d: Num. H-bond donors.

**Table 3:** Prediction of the pharmacokinetic properties of a selected compound.

	IA	SP	VDss	BBB	CYP2D6	CYP3A4	CYP1A2	CYP2C19	CYP2C9	CYP2 D6/3A4	TC
C41	92.98	−2.00	0.44	0.71	No	Yes	Yes	No	No	No	1.73
C22	96.22	−2.23	0.81	0.89	No	Yes	Yes	No	No	No	0.95
C27	94.67	−1.22	0.63	0.79	No	No	No	No	No	No	1.46
C40	93.84	−1.47	0.33	0.68	No	No	Yes	No	No	No	1.66
C33	94.49	−1.24	0.62	0.76	No	No	No	No	No	No	1.43
C72	96.88	−2.45	0.00	−0.14	No	No	Yes	No	No	No	0.01
C94	95.67	−2.21	0.62	0.88	No	Yes	Yes	No	No	No	0.97
C136	95.67	−2.21	0.62	0.88	No	Yes	Yes	No	No	No	0.97
C110	93.10	−2.16	0.30	0.56	No	No	No	No	No	No	1.03
C24	95.56	−1.27	0.63	0.80	No	No	No	No	No	No	1.44
C44	94.46	−1.98	0.88	0.82	No	Yes	No	No	No	No	0.80
C51	91.53	−1.51	0.36	0.66	No	No	No	No	No	No	1.75
C68	91.89	−1.48	0.37	0.65	No	No	No	No	No	No	1.74
C70	96.33	−2.06	0.30	0.52	No	Yes	Yes	Yes	No	No	0.42
C98	92.23	−1.85	0.49	0.58	No	No	No	Yes	Yes	No	1.03
C34	96.48	−1.56	0.67	0.81	No	No	No	No	No	No	1.19
C49	93.01	−1.76	0.42	0.61	No	No	No	Yes	No	No	1.36
C63	96.53	−1.63	0.67	0.81	No	Yes	No	No	No	No	1.19
C65	95.59	−1.43	0.54	0.72	No	No	No	No	No	No	1.42
C79	94.45	−4.01	0.51	0.75	No	No	No	No	No	No	1.54
C81	93.92	−1.21	0.55	0.82	No	No	No	No	No	No	1.81
C83	94.42	−1.62	0.48	0.71	No	Yes	No	No	No	No	1.64
C61	96.20	−1.77	0.37	0.62	No	No	No	No	No	No	1.19
C78	93.43	−1.46	0.68	0.78	No	Yes	No	No	No	No	1.22
C90	95.24	−2.09	0.68	0.85	No	Yes	Yes	No	No	No	0.94
C99	93.70	−2.36	0.29	0.63	No	No	No	No	No	No	1.15
C104	95.12	−1.80	0.08	0.57	No	No	No	No	No	No	0.59
C25	94.85	−1.58	0.65	0.73	No	No	No	No	No	No	1.09
C26	96.23	−1.68	0.86	0.86	No	Yes	No	No	No	No	1.18
C31	95.57	−1.70	0.64	0.82	No	Yes	Yes	No	No	No	1.17
C38	93.12	−1.77	0.28	0.63	No	Yes	No	No	No	No	1.31
C133	95.22	−1.86	1.11	0.55	No	Yes	Yes	No	No	No	1.21
C28	94.68	−1.74	0.51	0.66	No	No	No	No	No	No	1.28
C35	96.13	−1.46	0.69	0.77	No	No	No	No	No	No	1.18

IA: Intestinal absorption; SP: Skin Permeability; TC: Total clearance (log mL/min/kg).

**Table 4:** The calculated binding free energy for analyzed complexes using MM-GBSA.

Complexes	ΔG (kcal/mol)
2JK6-C41	−65.33
6UAK-C41	−55.00

**Table 5:** 2D Interactions of the Co-Crystallized Ligand in Proteins 6UAK and 2JK6.

*L. donovani* L.	*L. infantum* L.
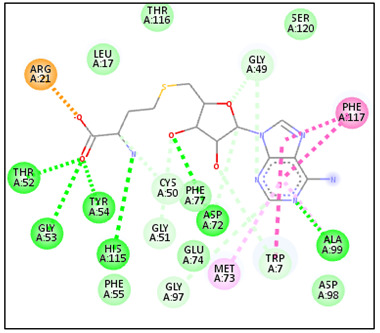	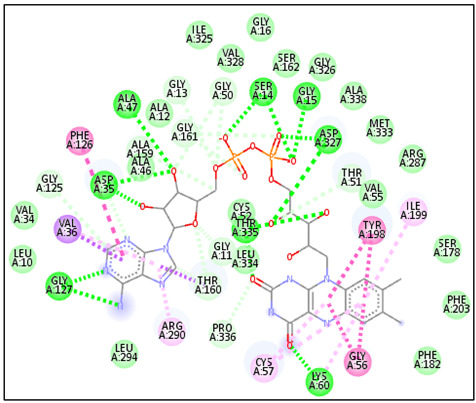

## Data Availability

Data are contained within the article.

## Supplementary Materials

The following supporting information can be downloaded at https://www.mdpi.com/article/10.3390/ph19050730/s1: Figure S1: Analysis of RMSD (A), RMSF (B), and protein–ligand contact histograms (C) for replicate molecular dynamics simulations of the 6UAK-C41 complex; Figure S2: Analysis of RMSD (A), RMSF (B), and protein–ligand contact histograms (C) for replicate molecular dynamics simulations of the 2JK6-C41 complex; Table S1: Docking scores of studied ligands against 6UAK and 2JK6. Table S2: Center Coordinates and Box Size Parameters (Å) of the Co-Crystallized Ligand in Proteins 6UAK and 2JK6.
