# Supplementary material for: Visceral Leishmaniasis: Integrated In Silico Screening of Djiboutian Medicinal Plant Phytoconstituents Targeting Leishmania donovani and Leishmania infantum
Source: Pharmaceuticals (Basel). 2026 May 6;19(5):730. doi: 10.3390/ph19050730 (PMC13209649; doi:10.3390/ph19050730)
Supplement: Supplementary file 1 [file pharmaceuticals-19-00730-s001.zip › pharmaceuticals-4213832-supplementary.pdf]

Table S1. Docking scores of studied ligands against 6UAK and 2JK6.

***L. donovani* L.**				***L. infantum* L**			
Compound	Score (kcal/mol)	Compound	Score (kcal/mol)	Compound	Score (kcal/mol)	Compound	Score (kcal/mol)
C41	-8.3	C80	-6.8	Liposomal amphotericin B	-8.6	C133	-6
C22	-8.2	C114	-6.8	C41	-7.5	C43	-6
C27	-8.1	C4	-6.7	Miltefosine	-7.3	C85	-6
C40	-8.1	C48	-6.7	C49	-7.2	C89	-6
C33	-8	C85	-6.7	C75	-7.2	C123	-6
C72	-8	C91	-6.7	C27	-7.1	C126	-6
C94	-8	C113	-6.7	C72	-7.1	C101	-6
C136	-7.9	C127	-6.7	C34	-7.1	C132	-6
C110	-7.8	C8	-6.6	C70	-7	C63	-5.9
C24	-7.7	C17	-6.6	C47	-7	C90	-5.9
C44	-7.7	C53	-6.6	C61	-6.9	C25	-5.9
C51	-7.7	C82	-6.6	C30	-6.9	C9	-5.9
C68	-7.7	C88	-6.6	C46	-6.9	C97	-5.9
C70	-7.7	C59	-6.5	C81	-6.8	C20	-5.9
C98	-7.7	C77	-6.5	C40	-6.7	C44	-5.8
C34	-7.6	C84	-6.5	C68	-6.7	C100	-5.8
C49	-7.6	C89	-6.5	C79	-6.7	C39	-5.8
C63	-7.6	C112	-6.5	C93	-6.7	C92	-5.8
C65	-7.6	C123	-6.5	C51	-6.6	C73	-5.8
C79	-7.6	C126	-6.5	C83	-6.6	C117	-5.8
C81	-7.6	C130	-6.5	C8	-6.6	C38	-5.7
C83	-7.6	C134	-6.5	C24	-6.5	C53	-5.7
C61	-7.5	C135	-6.5	C98	-6.5	C77	-5.7
C78	-7.5	C6	-6.4	C99	-6.5	C134	-5.7
C90	-7.5	C14	-6.4	C104	-6.5	C64	-5.7
C99	-7.5	C66	-6.4	C31	-6.5	C3	-5.7
C104	-7.5	C97	-6.4	C35	-6.5	C15	-5.7
C25	-7.4	C101	-6.4	C102	-6.5	C119	-5.7
C26	-7.4	C107	-6.4	C103	-6.5	C78	-5.6
C31	-7.4	C116	-6.4	C118	-6.5	C74	-5.6
C38	-7.4	C120	-6.4	C69	-6.5	C6	-5.6
C133	-7.4	C132	-6.4	C105	-6.5	C108	-5.6
C28	-7.3	C64	-6.3	C71	-6.5	C111	-5.6
C35	-7.3	C92	-6.3	C114	-6.5	C67	-5.5
Liposomal amphotericin B	-7.3	C96	-6.3	C82	-6.5	C4	-5.5
C21	-7.2	C115	-6.3	C21	-6.4	C120	-5.5
C36	-7.2	C125	-6.3	C10	-6.4	C115	-5.5
C128	-7.2	C3	-6.2	C86	-6.4	C125	-5.5
C10	-7.1	C15	-6.2	C58	-6.4	C16	-5.5
C29	-7.1	C16	-6.2	C62	-6.4	C1	-5.5
C42	-7.1	C57	-6.2	C88	-6.4	C13	-5.5
C45	-7.1	C1	-6.1	C33	-6.3	C131	-5.5
C47	-7.1	C5	-6.1	C94	-6.3	C87	-5.4
C76	-7.1	C18	-6.1	C136	-6.3	C19	-5.4
C86	-7.1	C60	-6.1	C26	-6.3	C32	-5.4
C30	-7	C73	-6.1	C80	-6.3	C124	-5.4
C74	-7	C109	-6.1	C113	-6.3	C12	-5.4
C75	-7	C117	-6.1	C42	-6.2	C84	-5.3
C93	-7	C122	-6.1	C76	-6.2	C60	-5.3
C100	-7	Miltefosine	-6.1	C37	-6.2	C55	-5.3
C102	-7	C32	-6	C59	-6.2	C7	-5.3
C103	-7	C108	-6	C112	-6.2	C17	-5.2

C118	-7	C111	-6	C130	-6.2	C96	-5.2
C121	-7	C119	-6	C135	-6.2	C57	-5.2
C37	-6.9	C129	-6	C107	-6.2	C5	-5.2
C39	-6.9	C20	-5.9	C116	-6.2	C109	-5.2
C43	-6.9	C124	-5.9	C65	-6.1	C122	-5.2
C46	-6.9	C12	-5.8	C28	-6.1	C129	-5.1
C58	-6.9	C13	-5.8	C36	-6.1	C2	-5.1
C69	-6.9	C54	-5.8	C29	-6.1	C66	-5
C87	-6.9	C55	-5.8	C45	-6.1	C54	-5
C105	-6.9	C131	-5.8	C121	-6.1	C23	-5
C9	-6.8	C7	-5.7	C11	-6.1	C14	-4.8
C11	-6.8	C2	-5.6	C50	-6.1	C18	-4.8
C19	-6.8	C56	-5.3	C48	-6.1	C56	-4.8
C50	-6.8	C106	-5.3	C91	-6.1	C106	-4.7
C62	-6.8	C23	-5.1	C127	-6.1	C52	-4.7
C67	-6.8	C95	-4.6	C22	-6	C95	-4.2
C71	-6.8	C52	5.6	C110	-6	C128	-4

Table S2. Center Coordinates and Box Size Parameters (Å) of the Co-Crystallized Ligand in Proteins 6UAK and 2JK6.

	***L. donovani* L**	***L. infantum* L**
**Center_x**	84.12	32.16
**Center_y**	41.06	55.04
**Center_z**	9.65	6.02
**Size_x**	20	30
**Size_y**	20	30
**Size_z**	20	30

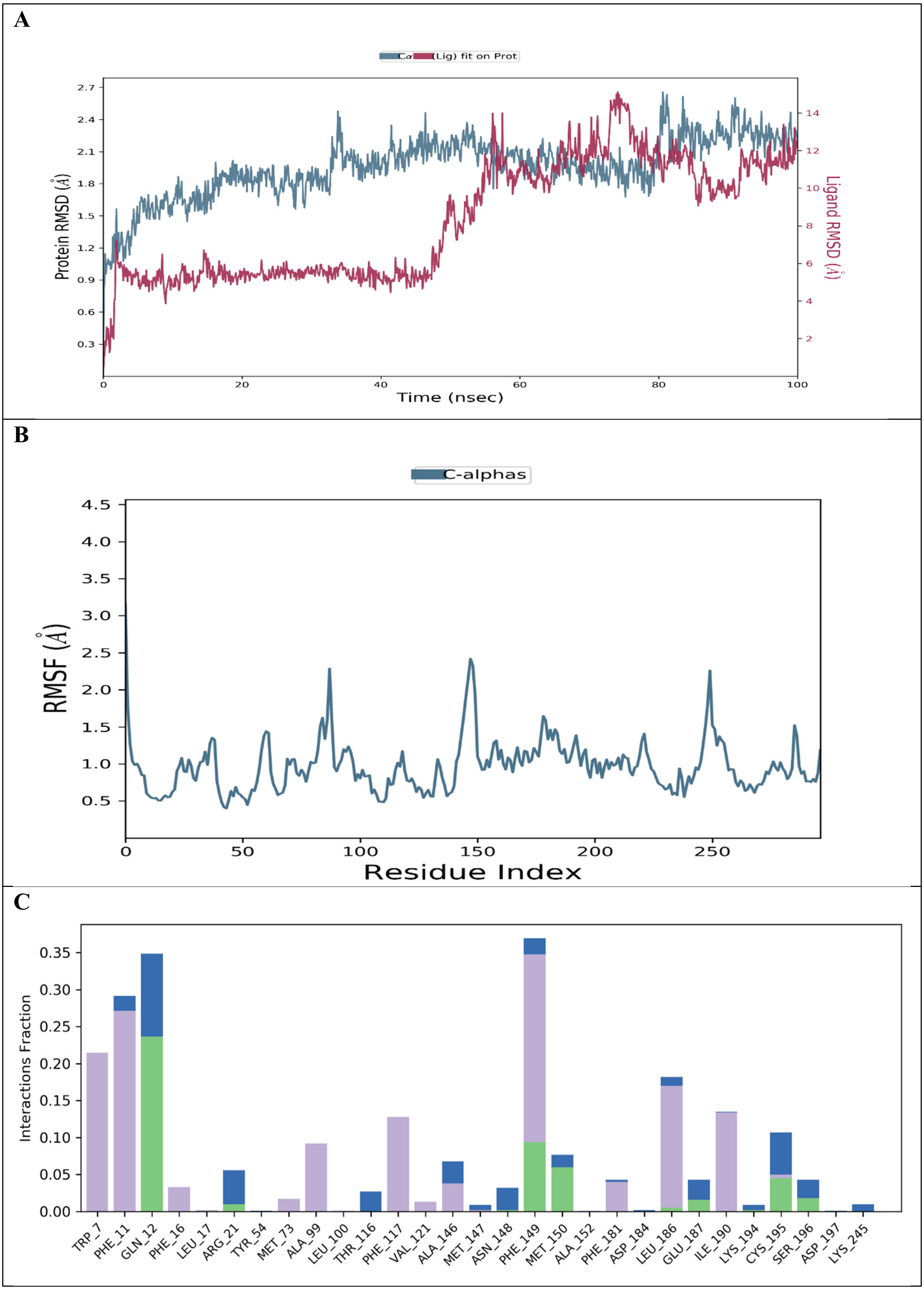

Figure S1: Analysis of RMSD (A), RMSF (B), and protein–ligand contact histograms (C) for replicate molecular dynamics simulations of the 6UAK–C41 complex.

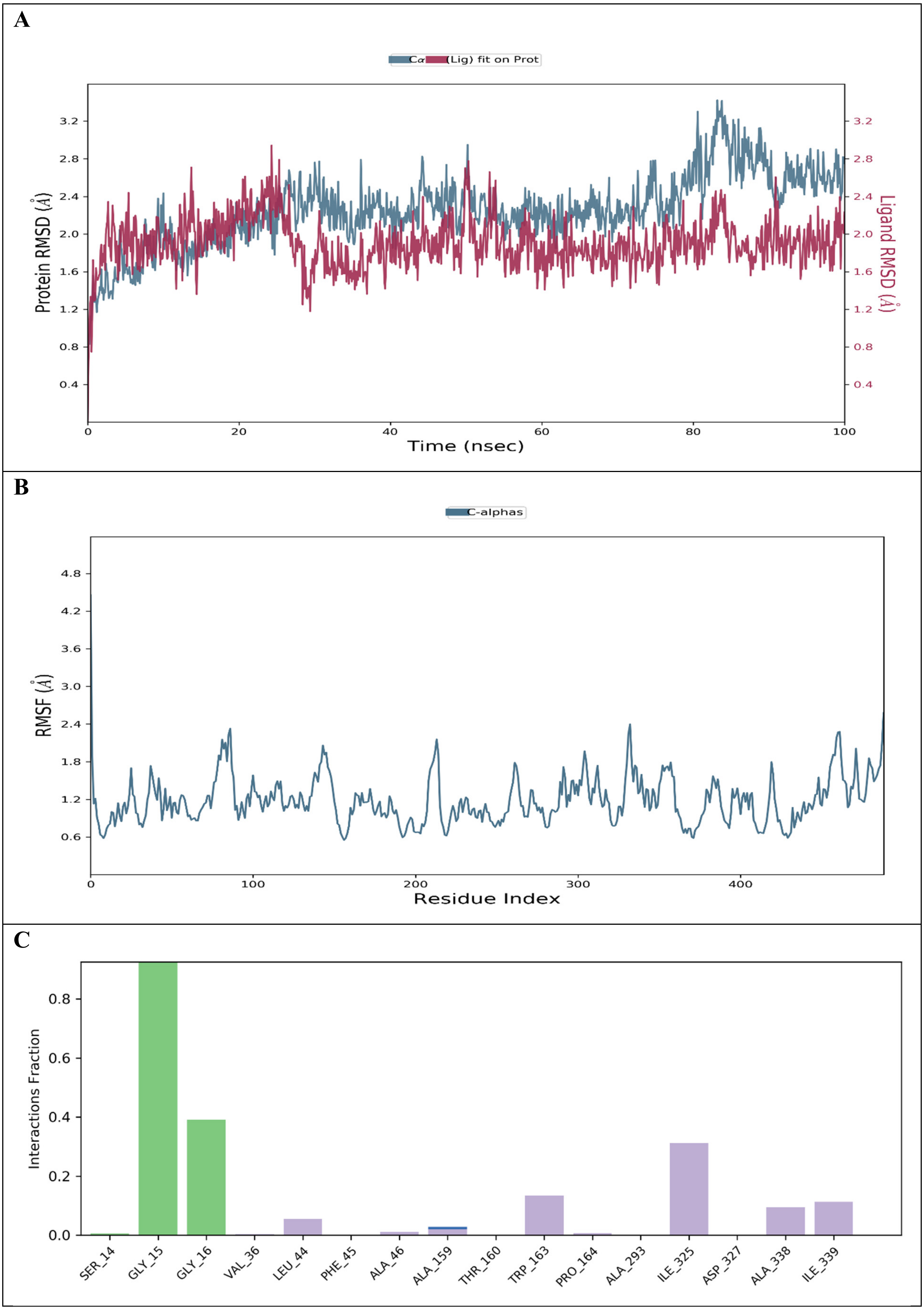

Figure S2: Analysis of RMSD (A), RMSF (B), and protein–ligand contact histograms (C) for replicate molecular dynamics simulations of the 2JK6–C41 complex.
